# Correction: Flexibility of a large blindly synthetized avatar database for occupational research: Example from the CONSTANCES cohort for stroke and knee pain

**DOI:** 10.1371/journal.pone.0324790

**Published:** 2025-05-19

**Authors:** Marc Fadel, Julien Petot, Pierre-Antoine Gourraud, Alexis Descatha

The Competing Interests statement for this article is incorrect. The correct Competing Interests statement is as follows: The authors have declared that they have competing interests: in addition to the affiliation, Alexis Descatha has received fees from Elsevier Masson as editor-in-chief of the journal “les archives des maladies professionnelles et de l’environnement”. PA Gourraud is the founder of Methodomics (2008) and the co-founder of Big data Santé (2018). He consults for major pharmaceutical companies, and start-ups, all of which are handled through academic pipelines (AstraZeneca, Amgen, Biogen, Boston Scientific, Cook, Docaposte, Edimark, Ellipses, Elsevier, Grunenthal, Janssen, IAGE, Lek, Methodomics, Merck, Mérieux, Octopize, Sanofi-Genzyme, Lifen, Aspire UAE). PA Gourraud is a volunteer board member at AXA not-for-profit mutual insurance company (2021). He has no prescription activity with either drugs or devices. He receives no wages from these activities. Octopize had no role in the design of the study, collection and analysis of data or decision to publish.
