# Supplementary material for: Flexibility of a large blindly synthetized avatar database for occupational research: Example from the CONSTANCES cohort for stroke and knee pain
Source: PLoS One. 2024 Jul 31;19(7):e0308063. doi: 10.1371/journal.pone.0308063 (PMC11290644; doi:10.1371/journal.pone.0308063)
Supplement: S1 Appendix — (PDF) [file pone.0308063.s001.pdf]

S1 Appendix: List of variables to which weights were assigned and Table of all raw/avatar pair odds ratios

- Age (continuous)
- Sex (binary)
- Borg (continuous)
- Work exposure “arm above the shoulder” (binary)
- Retirement age (continuous)
- Current shoulder pain (binary)
- Current elbow pain (binary)
- Current knee pain (binary)
- Current shoulder pain intensity (continuous)
- Current elbow pain intensity (continuous)
- Current knee pain intensity (continuous)
- Center for Epidemiologic Studies- Depression (CESD) scale (continuous)
- Daily limitations (categorial)
- Limitations for carrying 5kg (categorial)
- Most advanced certificate obtained (categorial)
- Occupational status (categorial)
- Body mass index (continuous)
- Personal history of stroke (binary)
- Personal history of myocardial infarction (binary)
- Long working hours exposure (continuous)
- Stroke (binary)
- Effort reward balance ratio (continuous)

S1 Appendix: Table of all raw/avatar pair odds ratios

Variables	Odds ratio Raw	Odds ratio Avatar
**All Stroke analysis**		
Age (Years)	1.07 (1.06-1.07)	1.07 (1.07-1.08)
Sex (male)	1.93 (1.66-2.25)	2.15 (1.85-2.51)
Body mass index (kg/m²)	1.05 (1.04-1.07)	1.10 (1.08-1.12)
Dyslipidemia	5.41 (4.63-6.30)	5.63 (4.81-6.57)
Smoking Pack.Years <30	1.07 (0.91-1.25)	1.19 (1.02-1.41)
Smoking Pack.Years ≥30	3.67 (2.90-4.60)	7.46 (5.58-9.82)
Diabetes	3.41 (2.34-4.79)	4.82 (3.24-6.89)
High blood pressure	4.52 (3.88-5.24)	4.40 (3.78-5.13)
Occupation Self-employed/CEO	1.16 (0.95-1.42)	1.15 (0.95-1.39)
Occupation Low-skilled	0.98 (0.78-1.22)	0.85 (0.68-1.06)
Occupation Blue-collar	1.73 (1.35-2.20)	1.41 (1.08-1.81)
Long working hours ≥10 years	1.72 (1.43-2.05)	1.53 (1.26-1.84)
**All Stroke analysis Male subgroup**		
Age (Years)	1.08 (1.07-1.09)	1.08 (1.07-1.09)
Body mass index (kg/m²)	1.05 (1.03-1.08)	1.09 (1.06-1.12)
Dyslipidemia	5.20 (4.30-6.27)	5.18 (4.28-6.25)
Smoking Pack.Years <30	1.19 (0.97-1.45)	1.21 (0.98-1.48)
Smoking Pack.Years ≥30	3.01 (2.26-3.96)	5.35 (3.75-7.46)
Diabetes	4.78 (3.15-6.94)	6.69 (4.32-9.87)
High blood pressure	4.65 (3.86-5.60)	4.85 (4.03-5.82)
Occupation Self-employed/CEO	1.18 (0.92-1.52)	0.98 (0.78-1.24)
Occupation Low-skilled	1.26 (0.90-1.73)	0.97 (0.71-1.31)
Occupation Blue-collar	1.38 (1.02-1.86)	1.02 (0.76-1.36)
Long working hours ≥10 years	1.65 (1.34-2.02)	1.34 (1.08-1.65)
**All Stroke analysis Female subgroup**		
Age (Years)	1.05 (1.04-1.06)	1.06 (1.05-1.07)
Body mass index (kg/m²)	1.04 (1.02-1.07)	1.10 (1.07-1.13)
Dyslipidemia	4.66 (3.49-6.14)	5.30 (3.97-7.00)
Smoking Pack.Years <30	0.77 (0.59-1.01)	1.02 (0.78-1.34)
Smoking Pack.Years ≥30	4.21 (2.71-6.27)	11.0 (6.41-17.7)
Diabetes	1.41 (0.50-3.08)	1.88 (0.57-4.44)
High blood pressure	3.71 (2.83-4.81)	3.05 (2.27-4.05)
Occupation Self-employed/CEO	0.82 (0.58-1.16)	1.08 (0.76-1.52)
Occupation Low-skilled	0.95 (0.70-1.30)	0.98 (0.71-1.35)
Occupation Blue-collar	1.88 (1.09-3.07)	1.54 (0.81-2.68)
Long working hours ≥10 years	1.14 (0.73-1.68)	1.04 (0.62-1.63)
**Stroke subtype analysis**		
Age (Years) ischemic	1.07 (1.06-1.08)	1.08 (1.07-1.09)
Age (Years) hemorrhagic	1.06 (1.04-1.07)	1.07 (1.06-1.08)
Sex ischemic	2.55 (2.12-3.07)	2.98 (2.45-3.63)
Sex hemorrhagic	0.99 (0.75-1.30)	1.08 (0.80-1.44)
Body mass index (kg/m²) ischemic	1.06 (1.05-1.08)	1.02 (0.99-1.05)
Body mass index (kg/m²) hemorrhagic	1.12 (1.09-1.14)	1.07 (1.02-1.11)

Dyslipidemia ischemic	6.66 (5.59-7.94)	6.50 (5.41-7.81)
Dyslipidemia hemorrhagic	2.83 (2.02-3.98)	3.81 (2.71-5.35)
Smoking Pack.Years <30 ischemic	1.10 (0.91-1.32)	1.23 (1.01-1.50)
Smoking Pack.Years <30 hemorrhagic	1.0 (0.74-1.34)	1.02 (0.73-1.42)
Smoking Pack.Years ≥30 ischemic	3.75 (2.86-4.90)	7.15 (5.06-10.1)
Smoking Pack.Years ≥30 hemorrhagic	3.46 (2.23-5.36)	8.54 (5.07-14.2)
Diabetes ischemic	4.61 (3.20-6.65)	7.10 (4.87-10.4)
Diabetes hemorrhagic	Not estimable	Not estimable
High blood pressure ischemic	5.01 (4.21-5.97)	4.86 (4.06-5.83)
High blood pressure hemorrhagic	3.37 (2.50-4.55)	3.78 (2.76-5.17)
Occupation Self-employed/CEO ischemic	1.23 (0.97-1.57)	1.17 (0.93-1.48)
Occupation Self-employed/CEO hemorrhagic	1.01 (0.71-1.45)	1.19 (0.82-1.75)
Occupation Low-skilled ischemic	1.13 (0.87-1.47)	0.85 (0.66-1.11)
Occupation Low-skilled hemorrhagic	0.66 (0.43-1.02)	0.83 (0.54-1.30)
Occupation Blue-collar ischemic	2.03 (1.52-2.70)	1.60 (1.19-2.15)
Occupation Blue-collar hemorrhagic	1.10 (0.67-1.82)	0.80 (0.42-1.51)
Long working hours ≥10 years ischemic	1.77 (1.43-2.18)	1.43 (1.13-1.81)
Long working hours ≥10 years hemorrhagic	1.60 (1.13-2.27)	1.76 (1.22-2.54)
**Stroke subtype analysis Male subgroup**		
Age (Years) ischemic	1.08 (1.07-1.09)	1.08 (1.07-1.10)
Age (Years) hemorrhagic	1.06 (1.04-1.08)	1.08 (1.05-1.10)
Body mass index (kg/m²) ischemic	1.06 (1.04-1.08)	1.10 (1.07-1.14)
Body mass index (kg/m²) hemorrhagic	1.03 (0.98-1.08)	1.03 (0.96-1.10)
Dyslipidemia ischemic	5.94 (4.82-7.31)	5.47 (4.42-6.77)
Dyslipidemia hemorrhagic	2.93 (1.86-4.64)	3.70 (2.32-5.88)
Smoking Pack.Years <30 ischemic	1.10 (0.88-1.38)	1.19 (0.94-1.51)
Smoking Pack.Years <30 hemorrhagic	1.56 (1.00-2.42)	1.43 (0.88-2.33)
Smoking Pack.Years ≥30 ischemic	3.0 (2.21-4.09)	5.71 (3.90-8.35)
Smoking Pack.Years ≥30 hemorrhagic	3.02 (1.56-5.83)	4.41 (1.81-10.7)
Diabetes ischemic	5.81 (3.88-8.71)	8.48 (5.57-12.9)
Diabetes hemorrhagic	Not estimable	Not estimable
High blood pressure ischemic	4.48 (3.64-5.51)	4.64 (3.75-5.73)
High blood pressure hemorrhagic	5.39 (3.59-8.09)	6.21 (4.08-9.44)
Occupation Self-employed/CEO ischemic	1.24 (0.92-1.65)	0.98 (0.76-1.28)
Occupation Self-employed/CEO hemorrhagic	1.01 (0.60-1.70)	1.12 (0.66-1.93)
Occupation Low-skilled ischemic	1.46 (1.02-2.08)	0.98 (0.69-1.37)
Occupation Low-skilled hemorrhagic	0.65 (0.29-1.45)	1.01 (0.49-2.05)
Occupation Blue-collar ischemic	1.53 (1.10-2.14)	1.03 (0.74-1.44)
Occupation Blue-collar hemorrhagic	0.93 (0.48-1.81)	0.71 (0.33-1.56)
Long working hours ≥10 years ischemic	1.53 (1.21-1.93)	1.25 (0.97-1.60)
Long working hours ≥10 years hemorrhagic	2.20 (1.43-3.38)	1.73 (1.09-2.76)
**Stroke subtype analysis Female subgroup**		
Age (Years) ischemic	1.05 (1.03-1.06)	1.06 (1.04-1.08)
Age (Years) hemorrhagic	1.05 (1.03-1.07)	1.06 (1.04-1.08)
Body mass index (kg/m²) ischemic	1.06 (1.03-1.09)	1.12 (1.07-1.16)
Body mass index (kg/m²) hemorrhagic	1.02 (0.98-1.05)	1.09 (1.03-1.14)

Dyslipidemia ischemic	6.08 (4.33-8.53)	7.09 (4.95-10.2)
Dyslipidemia hemorrhagic	2.83 (1.68-4.75)	3.94 (2.38-6.54)
Smoking Pack.Years <30 ischemic	0.85 (0.61-1.19)	1.07 (0.75-1.52)
Smoking Pack.Years <30 hemorrhagic	0.66 (0.42-1.02)	0.69 (0.43-1.13)
Smoking Pack.Years ≥30 ischemic	3.53 (1.97-6.30)	5.28 (2.12-13.2)
Smoking Pack.Years ≥30 hemorrhagic	Not estimable	Not estimable
Diabetes ischemic	2.20 (0.94-5.61)	4.22 (1.72-10.3)
Diabetes hemorrhagic	Not estimable	Not estimable
High blood pressure ischemic	5.18 (3.76-7.13)	4.35 (3.04-6.23)
High blood pressure hemorrhagic	1.97 (1.20-3.23)	1.96 (1.14-3.36)
Occupation Self-employed/CEO ischemic	0.69 (0.42-1.11)	0.96 (0.59-1.56)
Occupation Self-employed/CEO hemorrhagic	1.01 (0.61-1.69)	1.24 (0.72-2.15)
Occupation Low-skilled ischemic	1.15 (0.79-1.69)	1.09 (0.71-1.67)
Occupation Low-skilled hemorrhagic	0.67 (0.39-1.13)	0.78 (0.44-1.38)
Occupation Blue-collar ischemic	1.94 (1.01-3.75)	2.16 (1.08-4.33)
Occupation Blue-collar hemorrhagic	1.76 (0.78-3.99)	0.97 (0.29-3.21)
Long working hours ≥10 years ischemic	1.29 (0.78-2.13)	0.40 (0.15-1.08)
Long working hours ≥10 years hemorrhagic	0.89 (0.43-1.84)	1.81 (0.96-3.40)
**Knee pain analysis**		
Age (years) moderate	1.00 (1.00-1.00)	1.00 (1.00-1.00)
Age (years) severe	1.03 (1.02-1.03)	1.04 (1.04-1.04)
Sex moderate	0.91 (0.89-0.93)	0.91 (0.89-0.93)
Sex severe	0.78 (0.76-0.80)	0.74 (0.72-0.77)
Body mass index (kg/m²) moderate	1.02 (1.02-1.02)	1.04 (1.04-1.04)
Body mass index (kg/m²) severe	1.10 (1.10-1.10)	1.20 (1.20-1.21)
Occupation Self-employed/CEO moderate	0.88 (0.85-0.90)	0.86 (0.84-0.88)
Occupation Self-employed/CEO severe	0.68 (0.65-0.70)	0.56 (0.54-0.59)
Occupation Low-skilled moderate	0.99 (0.96-1.02)	1.03 (1.00-1.06)
Occupation Low-skilled severe	1.40 (1.35-1.46)	1.52 (1.46-1.59)
Occupation Blue-collar moderate	0.99 (0.95-1.04)	1.09 (1.05-1.14)
Occupation Blue-collar severe	1.82 (1.73-1.91)	2.07 (1.96-2.18)
Sedentary lifestyle moderate	0.88 (0.86-0.90)	0.95 (0.93-0.97)
Sedentary lifestyle severe	1.14 (1.11-1.17)	1.22 (1.18-1.26)
Borg moderate	1.25 (1.22-1.29)	1.32 (1.29-1.36)
Borg severe	1.77 (1.71-1.83)	1.86 (1.79-1.93)
**Knee pain analysis Male subgroup**		
Age (years) moderate	0.99 (0.99-0.99)	1.00 (1.00-1.00)
Age (years) severe	1.02 (1.02-1.02)	1.04 (1.03-1.04)
Body mass index (kg/m²) moderate	1.01 (1.01-1.02)	1.03 (1.03-1.04)
Body mass index (kg/m²) severe	1.10 (1.09-1.11)	1.20 (1.29-1.21)
Occupation Self-employed/CEO moderate	0.89 (0.85-0.93)	0.86 (0.82-0.89)
Occupation Self-employed/CEO severe	0.65 (0.61-0.69)	0.52 (0.49-0.56)
Occupation Low-skilled moderate	1.00 (0.95-1.06)	1.04 (0.99-1.09)
Occupation Low-skilled severe	1.19 (1.11-1.28)	1.40 (1.30-1.50)
Occupation Blue-collar moderate	1.07 (1.02-1.13)	1.13 (1.07-1.19)
Occupation Blue-collar severe	1.82 (1.71-1.94)	1.83 (1.71-1.96)

Sedentary lifestyle moderate	0.88 (0.85-0.91)	0.96 (0.93-0.99)
Sedentary lifestyle severe	1.15 (1.10-1.20)	1.24 (1.19-1.30)
Borg moderate	1.34 (1.28-1.39)	1.36 (1.31-1.42)
Borg severe	1.95 (1.85-2.04)	2.00 (1.89-2.11)
**Knee pain analysis Female subgroup**		
Age (years) moderate	1.00 (1.00-1.00)	1.00 (1.00-1.00)
Age (years) severe	1.03 (1.03-1.03)	1.05 (1.04-1.05)
Body mass index (kg/m²) moderate	1.03 (1.03-1.04)	1.06 (1.05-1.06)
Body mass index (kg/m²) severe	1.11 (1.10-1.11)	1.22 (1.22-1.23)
Occupation Self-employed/CEO moderate	0.90 (0.86-0.94)	0.89 (0.86-0.93)
Occupation Self-employed/CEO severe	0.75 (0.71-0.79)	0.64 (0.61-0.69)
Occupation Low-skilled moderate	0.96 (0.93-1.00)	1.01 (0.97-1.05)
Occupation Low-skilled severe	1.44 (1.38-1.51)	1.54 (1.46-1.62)
Occupation Blue-collar moderate	0.89 (0.82-0.98)	1.12 (1.03-1.22)
Occupation Blue-collar severe	2.20 (2.01-2.41)	3.44 (3.13-3.78)
Sedentary lifestyle moderate	0.88 (0.86-0.91)	0.95 (0.92-0.97)
Sedentary lifestyle severe	1.16 (1.12-1.21)	1.22 (1.17-1.27)
Borg moderate	1.17 (1.13-1.22)	1.29 (1.24-1.34)
Borg severe	1.64 (1.56-1.71)	1.78 (1.69-1.88)
**Limitations analysis**		
Limitations due to health problems moderate	1.73 (1.69-1.77)	1.82 (1.78-1.86)
Limitations due to health problems severe	5.48 (5.32-5.65)	6.79 (6.57-7.02)
Limitations due to pain moderate	1.80 (1.73-1.88)	1.89 (1.81-1.97)
Limitations due to pain severe	6.78 (6.51-7.07)	7.86 (7.52-8.23)
Limitations for climbing stairs moderate	1.83 (1.72-1.95)	1.91 (1.79-2.04)
Limitations for climbing stairs severe	11.8 (11.2-12.5)	12.5 (11.8-13.3)
Limitations for walking moderate	1.72 (1.61-1.84)	2.10 (1.96-2.26)
Limitations for walking severe	10.6 (10.0-11.2)	13.1 (12.3-14.1)
Limitations for carrying 5kg moderate	1.46 (1.39-1.53)	1.79 (1.70-1.89)
Limitations for carrying 5kg severe	5.47 (5.23-5.72)	8.50 (8.06-8.97)
**Limitations analysis Male subgroup**		
Limitations due to health problems moderate	1.69 (1.63-1.75)	1.77 (1.70-1.83)
Limitations due to health problems severe	5.61 (5.36-5.86)	6.65 (6.32-6.99)
Limitations due to pain moderate	1.78 (1.67-1.90)	1.83 (1.72-1.96)
Limitations due to pain severe	6.93 (6.52-7.38)	7.47 (6.98-8.00)
Limitations for climbing stairs moderate	1.68 (1.52-1.85)	1.70 (1.54-1.88)
Limitations for climbing stairs severe	11.1 (10.2-12.1)	10.8 (9.83-11.9)
Limitations for walking moderate	1.56 (1.40-1.73)	2.04 (1.82-2.29)
Limitations for walking severe	11.0 (10.1-12.1)	12.9 (11.6-14.4)
Limitations for carrying 5kg moderate	1.37 (1.23-1.54)	1.79 (1.60-2.01)
Limitations for carrying 5kg severe	7.47 (6.78-8.24)	10.2 (9.20-11.4)
**Limitations analysis Female subgroup**		
Limitations due to health problems moderate	1.74 (1.68-1.80)	1.84 (1.78-1.90)
Limitations due to health problems severe	5.29 (5.08-5.50)	6.74 (6.45-7.05)
Limitations due to pain moderate	1.80 (1.70-1.90)	1.92 (1.81-2.03)
Limitations due to pain severe	6.57 (6.22-6.94)	7.99 (7.53-8.49)

Limitations for climbing stairs moderate	1.92 (1.77-2.08)	2.05 (1.89-2.24)
Limitations for climbing stairs severe	12.0 (11.2-12.9)	13.4 (12.4-14.5)
Limitations for walking moderate	1.80 (1.65-1.96)	2.11 (1.92-2.32)
Limitations for walking severe	10.0 (9.32-10.8)	12.8 (11.7-14.0)
Limitations for carrying 5kg moderate	1.44 (1.36-1.51)	1.74 (1.64-1.85)
Limitations for carrying 5kg severe	4.86 (4.62-5.12)	7.63 (7.17-8.12)
