# Supplementary material for: Flexibility of a large blindly synthetized avatar database for occupational research: Example from the CONSTANCES cohort for stroke and knee pain
Source: PLoS One. 2024 Jul 31;19(7):e0308063. doi: 10.1371/journal.pone.0308063 (PMC11290644; doi:10.1371/journal.pone.0308063)
Supplement: Attachment — Submitted filename: Response.docx [file pone.0308063.s002.docx]

Journal Requirements:

1. Please ensure that your manuscript meets PLOS ONE's style requirements, including those for file naming. The PLOS ONE style templates can be found at

https://journals.plos.org/plosone/s/file?id=wjVg/PLOSOne_formatting_sample_main_body.pdf and

https://journals.plos.org/plosone/s/file?id=ba62/PLOSOne_formatting_sample_title_authors_affiliations.pdf

**R1.1. Files were reviewed and corrected to fit the PLOS ONE’s style requirements. File were renamed accordingly.**

2. We note that the grant information you provided in the ‘Funding Information’ and ‘Financial Disclosure’ sections do not match.When you resubmit, please ensure that you provide the correct grant numbers for the awards you received for your study in the ‘Funding Information’ section.

**R1.2. Grant information was updated in the submission system was updated to match the one in the manuscript. The funding information was removed from the manuscript accordingly to PLOS ONE’s requirements. Not all funding had specific grant numbers (notably for the funding of the CONSTANCES cohort).**

3. Thank you for stating in your Funding Statement:

"NO - Include this sentence at the end of your statement: The funders had no role in study design, data collection and analysis, decision to publish, or preparation of the manuscript.

Funding. The study is funded by a regional Public Fund (TEC-TOP project): Pays-de-la-Loire Region, Angers Loire Métropole, Univ Angers, CHU Angers. The CONSTANCES Cohort Study was supported and funded by the French National Health Insurance Fund (“Caisse nationale d’assurance maladie”, CNAM). The CONSTANCES Cohort Study is an “Infrastructure nationale en Biologie et Santé” and benefits from a grant from the French National Agency for Research (ANR-11-INBS-0002). CONSTANCES is also partly funded by Merck Sharp & Dohme (MSD), AstraZeneca, Lundbeck and L’Oréal through Inserm-Transfert. None of these funding sources had any role in the design of the study, collection and analysis of data or decision to publish."

Please provide an amended statement that declares *all* the funding or sources of support (whether external or internal to your organization) received during this study, as detailed online in our guide for authors at http://journals.plos.org/plosone/s/submit-now. Please also include the statement “There was no additional external funding received for this study.” in your updated Funding Statement.

Please include your amended Funding Statement within your cover letter. We will change the online submission form on your behalf.

**R1.3. We have updated the founding statement accordingly.**

4. In your Data Availability statement, you have not specified where the minimal data set underlying the results described in your manuscript can be found. PLOS defines a study's minimal data set as the underlying data used to reach the conclusions drawn in the manuscript and any additional data required to replicate the reported study findings in their entirety. All PLOS journals require that the minimal data set be made fully available. For more information about our data policy, please see http://journals.plos.org/plosone/s/data-availability.

Upon re-submitting your revised manuscript, please upload your study’s minimal underlying data set as either Supporting Information files or to a stable, public repository and include the relevant URLs, DOIs, or accession numbers within your revised cover letter. For a list of acceptable repositories, please see http://journals.plos.org/plosone/s/data-availability#loc-recommended-repositories. Any potentially identifying patient information must be fully anonymized.

Important: If there are ethical or legal restrictions to sharing your data publicly, please explain these restrictions in detail. Please see our guidelines for more information on what we consider unacceptable restrictions to publicly sharing data: http://journals.plos.org/plosone/s/data-availability#loc-unacceptable-data-access-restrictions. Note that it is not acceptable for the authors to be the sole named individuals responsible for ensuring data access.

We will update your Data Availability statement to reflect the information you provide in your cover letter.

**R1.4. Aggregated data of OR, are given in the S1 Appendix. Individual data are not available as open data due to the restriction by our national regulatory agency (“Commission nationale de l’informatique et des libertés”, n°910486). However, the CONSTANCES cohort is “an open epidemiological laboratory” and access to study protocols and data is available on justified request. We have updated the Data Availability statement.**

5. Your ethics statement should only appear in the Methods section of your manuscript. If your ethics statement is written in any section besides the Methods, please move it to the Methods section and delete it from any other section. Please ensure that your ethics statement is included in your manuscript, as the ethics statement entered into the online submission form will not be published alongside your manuscript.

**R1.5. The ethical statement was moved to the Methods section as instructed.**

6. Please upload a copy of Supporting Information Figure/Table/etc. Supplemental materials which you refer to in your text on page 4, 7 and 9.

**R1.6. We apologize for the oversight. Supplemental materials have been added. They contain a table with all the OR calculated for the study and the variables that had were given weight in the creation of the avatar dataset.**

Additional Editor Comments:

This is original research carried out to assess the utility of the 'Avatar' anonymization technique to create a synthetic data set that retains the statistical characteristics of the raw data set while preventing re-identification of individuals in the raw data set. The study is conducted well and the manuscript is an easy read. References 17 and 18 are important to get a better understanding of the Avatar methodology and the CONSTANCES database.

There are some minor edits that I will recommend. These include the following:

Page 3, paragraph 2, line 5: occupational ‘health research’ and rehabilitation

**R2.1. We have corrected this mistake.**

Page 3, paragraph 3, line 1: that ‘applies’ to occupational health

**R2.2. We have corrected this mistake.**

Page 3, paragraph 3, line 3: managed to “re-identify”

**R2.3. We have corrected this mistake.**

Page 3, paragraph 3, line 8: Possibly meant “properties” and not “proprieties”

**R2.4. We have corrected this mistake.**

Page 6 Table 1 title: Isn’t knee pain an outcome? It is labeled here as a risk factor

**R2.5. Actually, knee pain in this analysis is a risk factor since the models evaluated the association between having knee pain and the risk of daily life limitations (outcomes). For example, having moderate knee pain was associated with higher risk of limitations for carrying 5kg (OR = 1.79 in the avatar dataset and OR = 1.46 in the raw dataset).**

Page 7 second to last line: There is a reference for 'Supplemental Material', but no material has been provided that satisfies this reference. However, the major/important shortcoming in this version is that the supplemental material is missing. The authors mention "The dataset analyzed is available in the Supplemental materials" but I could not find the dataset. What was available included two .pdf documents:

2021-186 anglais

2021-186

Once the supplemental materials are received, we can proceed with the next step.

**R2.6. We apologize for the oversight. Supplemental materials have been added. They contain a table with all the OR calculated for the study and the variables that had were given weight in the creation of the avatar dataset.**

While revising your submission, please upload your figure files to the Preflight Analysis and Conversion Engine (PACE) digital diagnostic tool, https://pacev2.apexcovantage.com/. PACE helps ensure that figures meet PLOS requirements. To use PACE, you must first register as a user. Registration is free. Then, login and navigate to the UPLOAD tab, where you will find detailed instructions on how to use the tool. If you encounter any issues or have any questions when using PACE, please email PLOS at figures@plos.org. Please note that Supporting Information files do not need this step.

**R2.7. It seems I can’t connect to PACE (the login button doesn’t work for me?). We have change the Figure to Tiff.**
