# Supplementary material for: Flexibility of a large blindly synthetized avatar database for occupational research: Example from the CONSTANCES cohort for stroke and knee pain
Source: PLoS One. 2024 Jul 31;19(7):e0308063. doi: 10.1371/journal.pone.0308063 (PMC11290644; doi:10.1371/journal.pone.0308063)
Supplement: Attachment — Submitted filename: Response R2.docx [file pone.0308063.s003.docx]

Journal Requirements:

0.1. Please review your reference list to ensure that it is complete and correct. If you have cited papers that have been retracted, please include the rationale for doing so in the manuscript text, or remove these references and replace them with relevant current references. Any changes to the reference list should be mentioned in the rebuttal letter that accompanies your revised manuscript. If you need to cite a retracted article, indicate the article’s retracted status in the References list and also include a citation and full reference for the retraction notice.

**R0.1. The reference list has been checked.**

Reviewer #1. Thank you for the opportunity to review your manuscript titled "Flexibility of a large blindly synthesized avatar database for occupational research: example from the CONSTANCES cohort for stroke and knee pain."
Overall, the manuscript presents an intriguing study aiming to validate a large, blinded synthesized database. However, there are several points that require clarification and further elaboration:

1.1. The manuscript would benefit from a more detailed explanation of the statistical methods employed and a thorough interpretation of the results. It remains unclear whether the proposed method is superior to existing approaches, making it challenging to assess the validity of the conclusions drawn.

**R1.1. We understand the point raised by the reviewer, however, the aim of the paper was not to prove the superiority of the avatar method, but to test it in a specific context. Indeed, synthetic methods requires making assumptions on the distribution and the relation between variables of interest. In this work, we tried to create a synthetic dataset from a large database in the case where the team creating the synthetic dataset was blinded to the analyses that were done by the team in charge of the statistical analyses. Thus, this study represents a practical example of an exploratory analysis based on a cohort dataset with many variables and observations. Such example could be a first step and allow researchers and practitioners to make a first “crude” analysis on a dataset while minimizing the risk of reidentification in case of data leaking. A confirmatory analysis would of course have to be made on the real dataset, but would take less time thanks to the pre-analyses of the avatar dataset.**

**We have clarified the aim of the study in the discussion part of the manuscript:**

**p8 “The aim of the study was not to prove the statistical superiority of the avatar methods, but to test it in a blinded exploratory context of a large cohort dataset with minimum assumptions on the analyses that would be carried out”**

1.2. The data presented in Figure 1 indicate that the avatar odds ratio is above 2, suggesting a magnified effect. However, this aspect lacks explanation within the manuscript. Please provide further insights or discuss possible reasons for this observation.

**R1.2. At this stage of exploration of the avatar dataset, it is difficult to explain all of the**

**observed behaviors.** **One explanation could be linked to the behavior of the avatar method. By design, the Avatar method tends to center individuals to local barycenters.**

**With a parameter k=20, the avatar method could create some local clusters. This could**

**magnify the computed odds ratio.**

**We have added precision in the manuscript on this point p9.**

1.3. While the study mentions addressing privacy concerns in occupational health research, concrete examples of practical application are lacking. It would be beneficial to include specific instances or scenarios where the proposed approach can effectively address privacy issues in practice.

**R1.3. We have followed the reviewer’s advice and added practical example in the manuscript as well as details on the peculiarity of the occupational medical file.**

**In the introduction p3 “Likely, data related to the occupational medical files are of concern in terms of security and confidentiality as they deal with both health data (medical confidentiality) and data related to the workplace (professional confidentiality). Thus, the latest guidelines in France recommend collecting information on sensitive topics like medical fitness for work, occupational exposure but also addictions, psychiatric diseases, vaccinations etc”.**

**In the discussion p9 “Possible application examples could include restitution of statistical analyses to employers and workers on sensitive issues (like addictions), sharing databases used for analyses to employers and workers (transparency), sharing databases for multicentric analyses (like between occupational health units) with possibilities of international projects, Or sharing databases that were used to train machine learning models”.**

Reviewer #2: This paper is very important in the era of open access to medical data obtained in various centers. Conclusions drawn from such large datasets may be crucial for the development of e.g. new therapies, rehabilitation, etc, what Authors discussed in the Introduction. At the same time, the open access formula creates a risk of re-identification of individual data. Therefore, the use of the Avatar method has a great future, especially in the light of the results presented in this manuscript, which are worth publishing.
I just have a few minor questions and comments.

2.1. What does neighbors mean in the text, it is not clear for me

**R2.1. In the avatar methods, individual observations are projected into a complete multidimensional space. So, all individuals have numerical coordinates, and we can compute distances between individuals. Thus, by neighbors, we mean the closest individuals from a data point using the Euclidian distance in the projection space.**

**We have clarified this point in the methods p5: “****Neighbors correspond to the closest individuals from a data point using the Euclidian distance in the multidimensional projection space”.**

2.2. Please describe what the authors define as "long working hours" - how many hours a day, a week, a month?

**R2.2. Long working hours was defined in the CONSTANCES cohort as a** **working time greater than 10 hours a day for more than 50 days per year. The duration of exposure to long working hours was also collected.**

**We added this definition in the methods:**

**p5 “Long working hour was defined in the CONSTANCES cohort as working time greater than 10 hours a day for more than 50 days per year.”**

2.3 In the table 1 it is row: Current/former smoker <30 – no explanation whether 30 means e.g. 30 cigarette a day?

**R2.3. Smoking was quantified in pack-years. The information was added in the table.**

2.4. I don’t understand why wasn't the risk calculation for, Sedentary lifestyle and Borg scale. Although some authors deny the connection between sedentary lifestyle and stroke, many papers confirm it. Occupational physical effort may also be a risk factor for stroke. This current analysis could provide an additional argument for or against these relationships. If Authors don’t have a possibilities to perform additional analysis, they should explain the reasons
**R2.4. We agree with the reviewer that sedentary lifestyle and physical effort are relevant variables when studying stroke risk factors. However, this part of the analysis tried to reproduce results from a previously published study (Fadel et al. Association Between Reported Long Working Hours and History of Stroke in the CONSTANCES Cohort). Therefore, we included in this analysis only the variables that were used in the aforementioned study. The variables sedentary lifestyle and Borg scales were used in the second analysis which tried to identify risk factors for knee pain.**

2.5. In Discussion I found double use of „are more” (stealing data for economic gains are more are more frequent, and health facilities are no longer spared)

**R2.5. We have corrected the mistake.**
