# Supplementary material for: Flexibility of a large blindly synthetized avatar database for occupational research: Example from the CONSTANCES cohort for stroke and knee pain
Source: PLoS One. 2024 Jul 31;19(7):e0308063. doi: 10.1371/journal.pone.0308063 (PMC11290644; doi:10.1371/journal.pone.0308063)
Supplement: Attachment — Submitted filename: Response R4.docx [file pone.0308063.s004.docx]

Abstract:

Objectives:

Line 1: “Though the rise of big data in the field of occupational health…”

**R0.1. Corrected.**

Line 3: ‘…sensitive data from the field of …’

**R0.2. Corrected.**

Line 4: ‘…synthesized database developed from the CONSTANCES cohort by comparing associations between three independently selected outcomes, and various exposures.’

**R0.3. Corrected.**

Methods:

Line 2: ‘…(Octopize) that is agnostic to the primary or secondary data uses. Three main analyses of interest were chose to compare assocaitions computed in the raw and avatar dataset: risk of stroke (any stroke, and subtypes of stroke), risk of knee pain, and limitations associated with knee pain.’

**R0.4. Corrected.**

Results:

Line 2: ‘…more than 77% of the comparisons had….’

**R0.5. Corrected.**

Manuscript:

Introduction:

Page 3:

Paragraph 1, line 2. Remove the comma “,” after the word “Though”

**R0.6. Corrected.**

Paragraph 1, line 6: “sheer number of observations and variables available”

**R0.7. Corrected.**

Paragraph 2, line 6: “approaches need a substantial amount of data”

**R0.8. Corrected.**

Paragraph 2, line 7: “reproducible” and not “reproductible”

**R0.9. Corrected.**

Paragraph 2, Remove the sentence “Likely, data….vaccinations etc. [11, 12]” from paragraph 2 and “One important…sharing.” from paragraph 3.

**R0.10. Corrected.**

Paragraph 3:

Begin paragraph 3 as follows: “Issues regarding data security and possible re-identification have grown in the public and governmental agencies. Some studies managed to re-identify databases with great accuracy, which highlights the unsatisfactory reality for data anonymization [13,14]. Data related to the occupational medical files are of particular concern in terms of security and confidentiality as they deal with both health data (medical confidentiality) and data related to the workplace (professional confidentiality). Furthermore, the latest guidelines in France recommend collecting information on sensitive topics like medical fitness for work, occupational exposures, addictions, psychiatric diseases, vaccinations etc. [11,12]. With that in mind, the General Data Protection Regulation (GDPR) imposed harsh conditions for defining anonymous data and proposed several recommendations to evaluate the robustness of an anonymization process.[15]”

**R0.11. Corrected.**

Page 4:

Paragraph 1

line 4: “different from the original while retaining its statistical properties”

**R0.12. Corrected.**

line 5: “but they often require”

**R0.13. Corrected.**

line 6: “and rarely allow”

**R0.14. Corrected.**

line 7: “generating synthetic datasets”

**R0.15. Corrected.**

line 8: “and has proven to be effective in specific analysis in terms of statistical properties as well as ensuring data privacy”

**R0.16. Corrected.**

line 9: “in the context of a large cohort dataset.”

**R0.17. Corrected.**

Paragraph 2:

“The aim of this study was to create a large synthetic database and evaluate associations between three selected outcomes and several relevant variables.”

**R0.18. Corrected.**

Methods:

Line 3: “adults”

**R0.19. Corrected.**

Line 7: “…self-administered follow-up surveys.”

**R0.20. Actually, the questionnaire were all retrieved at the baseline inclusion.**

Line 7-9: “…examinations allowed capturing data on demographics, …… pain, and daily life limitations”.

**R0.21. Corrected.**

Page 5:

Paragraph 2:

Line 1: The word ‘in’ is used twice. Secondly, say, “both the raw dataset and the avatar dataset”

**R0.22. Corrected.**

Line 2: “were computed to evaluate the various associations considered”.

**R0.23. Corrected.**

Line 4: What is meant by number of pack years? Do you mean number of packs per year? If so, please state that more clearly.

**R0.24. Pack-years refer to the number of packs of cigarette a person has smoked per day per year. It is obtained by multiplying the mean number of packs smoked per day by the** **number of years the person has smoked (for example 1 pack/day smoked for 10 years corresponds to 10 pack-years).**

**This point was clarified in the methods:**

**P5. “(number of pack years, i.e. the number of packs smoked per day multiplied by the number of years the person has smoked)”**

Line 5: occupational status

**R0.25. Corrected.**

Line 6: white-collar jobs and not white jobs. This is mentioned twice in line 6.

**R0.26. Corrected.**

Line 14: “The third main analysis assessed the effect of knee pain (as defined in the 2nd main analysis) on several reported limitations: ….”

**R0.27. Corrected.**

Pag 6: Line 1: Remove the ellipses (…) after the word “diagnosed”

**R0.28. Corrected.**

Table 1 title:

“Distribution of Variables in the Raw and Avatar dataset, Crude Odds Ratios for the Occurrence of Stroke (Stroke Analysis), and Crude Odds Ratios for Reported Limitations, Across Selected Risk Factors”

**R0.29. Corrected.**

Table 1: Please state what is meant by Smoking (Pack.Years) more clearly. Does it mean packs per year?

**R0.30. A definition of pack-years was added in the method section.**

Table 1 footnote: OR for those with moderate knee pain; OR for those with severe knee pain.

**R0.31. Corrected.**

Page 8:

Paragraph 1:

The authors state “Though most of the number in each variable were statistically different….”, there is not statistical analysis reported to justify this statement. The only statement that can be made is that “Differences between the Avatar and Raw data sets in terms of the percentage distribution of responses for the various risk factors were mostly small (≤2%), with only one category…..10%).

**R0.32. As we do not show statistical tests, we have amended this part as recommended.**

Discussion:

Line 2 - 5: “The aim of this study was…..with minimum assumptions about the proposed analysis.”

**R0.33. Corrected.**

Line 6: “…for economic gain has become more frequent, and health facilities are not immune to this threat.”

**R0.34. Corrected.**

Page 9:

Line 2: “…exploratory analysis.”

**R0.35. Corrected.**

Line 3: researchers and not “searchers”.

**R0.36. Corrected.**

Line 5: “…relevant in the case of data pertaining to work, …”

**R0.37. Corrected.**

Line 6: “black-box machine learning…”

**R0.38. Corrected.**

Line 10: I don’t understand what is meant by “restitution of statistical analysis to employers and workers”

**R0.39. What we meant to say is that** **synthetize data can be presented to employers and workers without risking privacy breaches. This point was clarified in the discussion:**

**P9. “Possible application examples could include presentation of data to employers and workers on sensitive issues (like addictions), sharing databases used for analyses (transparency), sharing databases for multicentric analyses (e.g. across occupational health units) with possibilities of international projects, or sharing databases that were used to train machine learning models”**

Line 11: “…used for analysis with employers…”

**R0.40. Corrected (see R0.39).**

Line 12: “...multicentric analysis (e.g. across occupational health…)”

**R0.41. Corrected (see R0.39).**

Last paragraph: “The main limitation of this study…”

**R0.42. Corrected.**

Last line: “…which is not available in open access.”

**R0.43. Corrected.**

Page 10:

Line 1: “…was made since the aim of this study was to…”

**R0.44. Corrected.**

Line 2-3: “Most studies compare the overall of confidence intervals,[24] though this method has limitations in the case of large datasets with small confidence intervals, as was the case our analysis of knee pain, which had close OR with non-overlapping confidence intervals.”

**R0.45. Corrected.**

Line 9: “…from rare events like stroke, to common…”

**R0.46. Corrected.**
